# Therapeutic Alliance: A Comparison Study between Adolescent Patients and Their Therapists

**DOI:** 10.3390/ijerph182111238

**Published:** 2021-10-26

**Authors:** Vera Gergov, Mauri Marttunen, Nina Lindberg, Jari Lipsanen, Jari Lahti

**Affiliations:** 1 Adolescent Psychiatry, Helsinki University Hospital, University of Helsinki, 00260 Helsinki, Finland; mauri.marttunen@helsinki.fi; 2 Department of Psychology and Logopedics, Faculty of Medicine, University of Helsinki, 00290 Helsinki, Finland; jari.lipsanen@helsinki.fi (J.L.); jari.lahti@helsinki.fi (J.L.); 3 Mental Health Unit, National Institute for Health and Welfare, 00300 Helsinki, Finland; 4 Forensic Psychiatry, Helsinki University Hospital, University of Helsinki, 00260 Helsinki, Finland; nina.lindberg@hus.fi

**Keywords:** adolescents, psychotherapy, alliance, naturalistic study, predictors

## Abstract

The aim of this study was to examine the congruence of adolescent- and therapist-rated therapeutic alliance, and to explore which rating or combination of ratings would predict treatment outcome or premature termination. We also studied whether the alliance changes over the course of treatment and if the change is related to the outcome or dropout. This study comprised 58 adolescents clinically referred for psychotherapeutic interventions. The alliance (Working Alliance Inventory, patient/therapist ratings) and treatment outcomes (Beck Depression Inventory, Clinical Outcomes in Routine Evaluation—Outcome Measure) were measured at baseline and at 3-, 6-, and 12-month follow-ups. The alliance did not change significantly over the course of therapy, but adolescent and therapist ratings did not correlate. Low values in the early assessment of adolescent-rated alliance and discrepancy between the ratings were significant predictors of undesirable treatment outcome. Weak adolescent- or therapist-rated alliance later in treatment and change for the worse in adolescent-rated alliance was associated with treatment dropout. As adolescent-rated alliance predicts treatment outcome better than therapist-rated alliance, therapists should frequently use assessments of therapeutic relationship within the therapy and pay attention if the adolescent feels the alliance is weakening or his/her evaluation is contrary to the therapist’s.

## 1. Introduction

Mental health problems increase substantially in adolescence. Approximately 50% of lifetime diagnosed mental health disorders manifest by the age of 14, and the amount increases to 75% by the age of 24 [1] Psychotherapeutic interventions are effective among adolescents for treating mental disorders [2,3]. However, no treatment has proven to be clearly superior, and it is not yet known which adolescents benefit most from which type of treatment [4,5]. In order to find out the real-life effectiveness of psychotherapy, researchers have in recent years encouraged studies of clinically referred youths and of treatments delivered in clinical service settings [3]. As adolescents might not be as cognitively and verbally as advanced as adults in expressing their thoughts and feelings, adolescents commonly receive treatments that combine different treatment approaches and include also nonverbal components [6]. In addition, the therapists usually need to be more active and take a more directive role in the treatment of adolescents [7] in order to gain their trust and form a confidential relationship with the adolescent.

According to the so-called common factors model [8,9,10], there are factors that affect the treatment outcome regardless of the treatment modality. Common factors that have been found to have the largest effects include alliance, empathy, treatment expectations, cultural adaptation of the treatment, and therapist effects [10]. Alliance between the patient and the therapist has been described as the most important common factor related to good treatment outcome, despite the length or modality of the treatment [11,12,13,14]. A commonly used conceptualization of alliance in psychotherapy research literature is based on Bordin’s pantheoretical three-dimensional model [15]. The model defines working alliance as the therapist–patient collaboration on the goals and tasks of treatment during the therapy process, and the bond developed between them to work toward the agreed goals. The development of a “good enough” alliance early in therapy has been shown to be vital for the success of therapy, as it prevents patients from prematurely terminating treatment and enables a collaborative working space [16].

The assessment of therapeutic alliance can be made by the patient, by the therapist, or by both of them. In adult patients, the evaluation of the alliance by patients and therapists strongly correlates [17], and the alliance–outcome effect size is similar across different treatment approaches and alliance perspectives (e.g., patient and therapist) [11,13]. Alliance had a medium-sized effect (r = 0.28) on outcome in a recent meta-analysis of 295 studies with adult patients [11].

Alliance seems to develop by the third to fifth treatment session [18]. Alliance is typically assessed at the beginning of treatment, and low scores are regarded as a warning sign of later patient dissatisfaction or possible dropout and/or undesired treatment outcome [5]. However, according to a meta-analysis by Flückiger et al. (2018), the relation between alliance and treatment outcome seems to be weaker when alliance is measured early in the treatment compared with later evaluations [11].

Research on alliance between adolescent patients and their therapists is still scarce [19,20] and there is large methodological variability between the studies, e.g., studying a wider set of relational variables instead of focusing on alliance, using different alliance measurements or information sources, and variety of the timing of alliance measurement [21,22,23]. Yet, therapeutic alliance has been found to be an important treatment outcome predictor also with adolescents [19,22,24,25]. Studies on youth alliance have failed to fully support the three-factor model, suggesting that features of the alliance are less differentiated at a younger age, and instead propose a single-factor solution [26,27,28,29]. This might be explained by developmental differences between youth and adult patients, and common discrepancies among the goals of therapy set by adolescents, caregivers, and therapists [25]. According to a meta-analysis by Karver et al. (2018), the alliance–outcome effect sizes are comparable to those reported in adult patients (r = 0.19) [24]. A meta-analysis by Murphy and Hutton (2018) noted a higher effect size (r = 0.34) when the sample was limited to 12- to 19-year-old adolescents [19].

Studies with adolescents show that alliance ratings already during the first two therapy sessions are related to retention and outcome [30,31]. Maintenance of positive treatment alliance over time predicts successful outcome [29]. However, up until lately, we have had very little evidence on how the alliance develops and changes over time especially with adolescents [25,29], as most studies measure alliance only at the beginning of therapy. There are contradictory findings on whether the alliance measured at the beginning of treatment or that measured later predicts the outcome most strongly [32,33]. Shirk and Karver (2003) [33] and McLeod (2011) [23] noted that late measures of therapeutic relationship showed larger associations with treatment outcome than early alliance measures, but as later measurements are potentially confounded with symptom improvement, early alliance assessment is preferred [23]. In addition, there are several studies suggesting that it is, in fact, the change in alliance that best predicts the treatment outcome or treatment completion [21,34,35,36]. Bickman et al. (2012) found that therapeutic alliance had a small positive change over time regardless of informant, and youths with improving alliance also had more improvement in psychopathology ratings [21].

An ability to form and retain a positive working alliance might be even more crucial with adolescents than with adults, as adolescents are likely to have a more limited perception of psychotherapy and are typically referred for treatment by others [27,29]. External referral for therapy might lead to disagreements on the core components of alliance, agreement on the tasks, and goals of treatment [36]. In the meta-analysis by de Haan et al. (2013), poor therapeutic alliance was an important predictor of premature termination of treatment in youth psychotherapy [37]. O’Keeffe et al. (2020) also noted that ruptures in therapeutic alliance, especially if unsolved, are important warning signs of possible premature termination of treatment with adolescents [36].

Adolescents report improvement in psychotherapy more frequently than their parents or therapists [38], and yet adolescents are not always the central informants about their own treatment. The same goes for measuring alliance—it has not been very common in clinical practice or in research to measure, or even frequently ask the adolescents about, the alliance. Even rarer is to include both youth and therapist evaluations of the therapeutic alliance in the same study [5]. In previous studies on youth psychotherapy, the association between alliance and outcome was found to be stronger if the alliance is rated by therapists rather than by young patients [23,33]. However, in the meta-analysis by Karver et al. (2018) [24], the alliance perspective did not moderate the relation, and recent findings from Cirasola et al. (2021a) [22] suggest that both adolescent and therapist early ratings of the alliance are associated with symptom change. Yet, agreement between adolescent and therapist evaluation of alliance has previously been low or nonsignificant [19,24]. The association between alliance and outcome has been stronger if the informant is the same for both variables [23]. Discrepancy between adolescent and parent ratings has been associated with premature termination of treatment [30], but to our knowledge, the combination of adolescent- and therapist-rated alliance has not been tested as a predictor in studies other than that of van Benthem et al. (2020) [5]. That study showed that including the alliance ratings from both perspectives provided a stronger predictor of treatment outcome than using only one perspective. Youths with a strong alliance according to both perspectives had more favorable treatment outcome compared with youths with weaker alliance according to both perspectives.

The aim of this study was to examine the congruence of adolescent- and therapist-rated alliance, and to explore which rating or combination of ratings would be the most informative predictor of treatment outcome or premature termination of treatment. In addition, we aimed to study whether the alliance ratings change over the course of treatment and if the change is related to outcome or dropout. Based on previous findings, we hypothesized that there is low correlation between alliance rated by the adolescents and by the therapists, and that therapist-rated alliance would be a better predictor of treatment outcome. We also expected that alliance ratings would be quite stable with only a small increase over the course of therapy but that a later rating would be a better predictor of outcome. In addition, we hypothesized that change for the worse in the perceived alliance would predict worse treatment outcome and premature termination of treatment, as would discrepancy in the alliance ratings between adolescents and therapists.

## 2. Materials and Methods

### 2.1. Procedure

This study was conducted at the Division of Adolescent Psychiatry in the Department of Psychiatry, Helsinki University Hospital, Finland. The study is part of a larger study on the effectiveness and predictors of treatment outcome for psychotherapeutic interventions for adolescent psychiatric patients delivered in a naturalistic setting.

The adolescents were referred for psychotherapeutic interventions by secondary care psychiatric clinics for adolescents during the 2-year patient recruitment period (1 February 2012 to 31 January 2014). The study was conducted as part of ordinary outpatient care with the therapies conducted by private practitioners but overall treatment responsibility remaining with the psychiatric clinic which referred the patient for the index therapy. The participants filled in the self-assessment forms at baseline, which was after completing the assessment period for therapy (1 to 3 appointments), and again after 3, 6, and 12 months of treatment. The therapists evaluated the treatment alliance at the same time points. The study procedure and the results on the effectiveness of the interventions have been published in Gergov et al. (2015, 2021) [39,40].

### 2.2. Ethics

The study was accepted by the Ethics Committee of the Helsinki and Uusimaa Hospital District (276/13/03/03/2011). Permission to conduct the study was granted by the pertinent institutional authorities of Helsinki University Hospital (704/13/2011). All participants and their legal guardians provided written informed consent to participate after receiving verbal and written information about the study. Refusal did not affect the treatment the adolescents received.

### 2.3. Participants

The participants were 13- to 15-year-old adolescents (mean age 14.22 years, standard deviation 0.73) referred for psychotherapeutic interventions from secondary care psychiatric clinics in the region. There were no exclusion criteria for the study. In the study, 70.7% (*n* = 61) of the adolescents meeting the criteria agreed to participate in the study; 59 adolescents started the intervention; and 58 filled in the self-assessment at baseline. At the 3-month follow-up, no treatment or research dropout had occurred; at the 6-month follow-up, five adolescents had dropped out from treatment and two from the study; and at the 12-month follow-up, a total of 10 adolescents had dropped out from treatment and three had refused to continue participation in the study.

Psychiatrists responsible for the patients’ care assessed the psychiatric diagnoses using the ICD-10 classification [41]. The most typical diagnostic groups were F40–49: Neurotic, stress-related, and somatoform disorders (43.1%), F30–39: Mood disorders (27.6%), and F90–98: Behavioral and emotional disorders (20.7%). The sociodemographic variables were reported by the clinicians or verified from medical records. The sample characteristics did not differ significantly from the average patient population receiving publicly funded psychotherapeutic interventions in the region [39]. Sample characteristics are presented in more detail in Gergov et al. (2021) [40].

### 2.4. Treatment

There were 47 therapists participating in the study, representing various psychotherapeutic approaches. All therapists were trained and certified for the form of therapy they provided and were accepted as health-care practitioners by national authorities. The interventions included psychotherapy based on verbal interaction (*n* = 37, 63.8%; psychodynamic (*n* = 22), cognitive (*n* = 5), crisis- and trauma-focused (*n* = 3), family (*n* = 7)) and art and occupational therapies that also utilize nonverbal interaction (*n* = 21, 36.2%; music (*n* = 10), art (*n* = 5), occupational (*n* = 4), riding therapy (*n* = 2)). As there was no statistically significant difference in the outcomes between the two treatment arms in our previous study [40], the treatments were combined for this study; 81.0% of the adolescents received individual therapy, 12.1% family therapy, and the rest (6.9%) received group therapy. As the study was naturalistic, no standard treatment protocol was demanded. A total of 38 therapists treated one patient each, seven therapists treated two, one therapist treated three, and one treated four patients. Based on the intraclass correlation coefficient (ICC), therapist effect did not significantly explain variation in treatment outcome measures (ICC: 0.00–0.06).

### 2.5. Measures

#### 2.5.1. Working Alliance Inventory

The Working Alliance Inventory (WAI) [17] is a self-report questionnaire measuring the quality of the therapeutic relationship and agreement on goals and tasks in treatment. The measure is based on Bordin’s pantheoretical conceptualization of the alliance [15]. It consists of 36 items that are rated on a 7-point Likert scale, and a total score is counted for all items, ranging from 36 to 252. The WAI includes three scales: bond between the patient and therapist (12 items), agreement between the patient and therapist on the goals of therapy (12 items), and agreement between the patient and therapist on the tasks in therapy (12 items). The studies indicate that the WAI is unidimensional in adolescents [24,26,27,28] so the subscale scores were not used in this study. The WAI was originally created for adult therapy, but it has been widely used with adolescents [19,24] even though adolescent modifications are often made and a shorter version (WAI-S) [42] has been used more frequently and has been found to have good internal consistency with youth samples [24]. In this study the working alliance was rated by both patients (WAI-P) and therapists (WAI-T). As the short forms modified for adolescents were not available in Finnish when the study was conducted, the Finnish translation of the original 34-item inventory translated for the Helsinki Psychotherapy Study [43] was used. The internal consistency (Cronbach’s alpha, α) of the questionnaires was excellent in this study (WAI-P: α = 0.94; WAI-T: α = 0.95).

#### 2.5.2. Beck Depression Inventory

To assess depressive symptoms, participants filled in the Beck Depression Inventory (BDI-21) [44]. In the BDI-21, each of the 21 items is scored from 0 to 3, and the items are summed for a total score ranging from 0 to 63. The total score reflects the level of depression: 0–9 indicates a non-depressive mood, 10–15 mild depression, 16–23 moderate depression, and 24–63 severe depression. The BDI-21 has shown good psychometric properties [45] and is widely used in outcome studies with adolescents. The internal consistency was excellent (α = 0.95).

#### 2.5.3. Clinical Outcomes in Routine Evaluation—Outcome Measure

The Clinical Outcomes in Routine Evaluation—Outcome Measure (CORE-OM) [46] is a self-report questionnaire measuring psychological distress. Patients evaluate 34 statements using a 5-point Likert scale from 0 to 4, with 4 being the highest level of severity. The CORE-OM includes four subscales: subjective well-being (4 items), problems/symptoms (12 items), life functioning (12 items), and risk/harm (6 items). The score for each scale is the mean score of the items, and the total score is the mean across all the items (between 0 and 4). The CORE-OM has shown good psychometric properties and is among the most used measurements to assess change in psychotherapy [46,47]. The Finnish translation used for this study has been well accepted and its reliability and validity have been good especially in the clinical population [48]. In this study, the internal consistency of the questionnaire was also excellent (α = 0.96). A youth version of the CORE measure (The Young Person’s CORE, YP-CORE) has been published [49], but the Finnish translation [50] was not available at the beginning of our study.

### 2.6. Data Analyses

We used SPSS version 26 for statistical analyses. Data inspection revealed that all the assumptions (e.g., a normal distribution) of tests used were met. We assessed the relation between the rating of working alliance between the patients and the therapists with the paired samples *t*-test and measured the correlations at different time points with Pearson’s correlation, r. We used linear mixed models to examine the associations of baseline and 3-month adolescent-rated therapeutic alliance (WAI-P) and therapist-rated alliance (WAI-T) with the change in the BDI-21 and CORE-OM during the 12-month treatment.

We formed a variable describing the discrepancy in the alliance rating between adolescent and therapist (therapist rating minus adolescent rating) at baseline and at the 3-month follow-up according to Robbins et al. (2003) [30], who used a similar score as an indicator of an unbalanced alliance between adolescents and parents. In order to look at the discrepancy of the alliance ratings in more detail, the median split of the adolescent-rated (WAI-P) and the therapist-rated (WAI-T) values (Table 1) was used and four subgroups were distinguished similarly to those in van Benthem et al. (2020) [5], with alliance rated as (1) “weak” by both adolescent and therapist, (2) “weak” by adolescent and “strong” by the therapist, (3) “strong” by adolescent and “weak” by the therapist, and (4) “strong” by both adolescent and therapist.

We predicted treatment outcome in the first 12 months in separate linear mixed models with (1) the level of reported alliance at baseline rated by adolescent or therapist; (2) the level of reported alliance at 3-month follow-up rated by adolescent or therapist; (3) the change occurring in the adolescent- and therapist-rated alliance in the first three months; (4) the discrepancy between the adolescent- and therapist-rated alliance at baseline; (5) the discrepancy between the adolescent- and therapist-rated alliance at 3-month follow-up; (6) the subgroup of congruence of the adolescent–therapist alliance rating at baseline; and (7) the subgroup of congruence of the adolescent–therapist alliance rating at 3-month follow-up. The same was done for predicting treatment dropout with logistic regression analysis. For analysis with alliance congruence subgroup category as independent variable to predict treatment outcome, we used the fourth category (strong–strong) as the reference category. Considering the dropouts, statistical significance was determined based on 5000 bootstrapped, bias-corrected resamples.

The possible confounding effects of age, gender, and psychotropic medication were controlled in the predictor analyses for outcome. Three adolescents refused to continue their participation in the study during the 12-month follow-up period and we excluded them from the dropout prediction analysis.

The level of significance was defined as *p* < 0.05. The Pearson correlation (r) was interpreted as small = 0.1, moderate = 0.3, and strong = 0.5 [51]. We report effect sizes by using marginal R^2^ for all fixed effects [52,53,54]. Effect size estimation was done with R software version 3.5.1 [55] using the MuMIn package [56]. The magnitude of the effect size was interpreted as “small,” “medium,” and “large” with cutoff points of 0.02, 0.13, and 0.26, respectively [51]. Odds ratios (OR) were converted to R^2^ according to Lenhard and Lenhard (2016) [57]. Power calculations for linear mixed models were carried out by simulation (500 simulations per analysis) in R software version 4.0.3 [58] using simr package [59]. Using the available sample size of 58, large effect sizes could be detected as statistically significant with 80% power. We calculated observed power as a benchmark for future research, and it ranged from 11 to 99% for statistically significant results and from 0 to 72% for insignificant results. When evaluating treatment dropout using logistic regression analysis based on a priori power analysis with G * Power version 3.1.9.2 software [60], medium effect sizes (OR > 3.5) could be detected as statistically significant with the sample size of 58. We also calculated observed power as a benchmark for future research. It ranged from 69 to 93% for statistically significant results and from 0 to 39% for insignificant results.

## 3. Results

### 3.1. Congruence in Alliance Ratings of Patients and Therapists

The mean levels of perceived therapeutic alliance reported by patients (WAI-P) and therapists (WAI-T) did not significantly differ from each other at baseline (*t*(52) = −1.67, *p* = 0.10), 3-month follow-up (*t*(54) = −0.85, *p* = 0.40), 6-month follow-up (*t*(44) = −1.11, *p* = 0.28), or 12-month follow-up (*t*(37) = −1.16, *p* = 0.25) (Table 1). However, adolescent–therapist correlations at each time point were low: r = 0.09 at baseline, r = 0.24 at 3-month follow-up, r = 0.16 at 6-month follow-up, and r = 0.13 at 12-month follow-up. In the linear mixed effect models, overall association between adolescent and therapist alliance measures was positive and statistically significant (*p* = 0.02, r^2^ = 0.04), but the association did not change in time. Based on the ICC, the therapist effect did not significantly explain the variation in therapist-rated alliance.

### 3.2. Change in Perceived Therapeutic Alliance over the Course of Therapy

There was no statistically significant change in the alliance scores reported by the adolescents (WAI-P) over the course of therapy compared with the baseline assessment (*p* = 0.78). For the therapist assessment (WAI-T), there were also no statistically significant differences in the alliance scores at different time points (*p* = 0.14). The ratings of patients at different time points were significantly correlated (*p* < 0.02, r = 0.38 to 0.55) as were the ratings of the therapists (*p* < 0.001, r = 0.69 to 0.73). Correlation between adolescent- and therapist-rated alliance at each time point and change in alliance across time points showed a similar pattern in a sample with complete data from all time points (*n* = 34).

### 3.3. Predicting Treatment Outcome with Alliance Measured at Baseline and at 3-Month Follow-Up, and Change in Alliance in the First Three Months of Treatment

Lower WAI-P at baseline was associated with higher ratings in all outcome measures: BDI-21 (*p* = 0.02), CORE-OM total score (*p* < 0.01), CORE-OM well-being (*p* < 0.01), CORE-OM problems/symptoms (*p* < 0.01), CORE-OM life functioning (*p* < 0.01), and CORE-OM risk/harm (*p* = 0.05) (Table 2). The effect sizes were mostly in a medium range (R^2^ = 0.12 to 0.29). Time did not have a significant main effect in the analysis for any of the outcome measures, and there were no significant time × alliance interactions. The same effect was found already at baseline; for example, the patients who had lower BDI-21 scores rated the alliance significantly better.

When looking at the patient alliance ratings after receiving three months of treatment, lower WAI-P was associated with higher scores in CORE-OM total score (*p* = 0.03), CORE-OM well-being (*p* = 0.02), CORE-OM problems/symptoms (*p* < 0.01), and CORE-OM life functioning (*p* = 0.02) with medium effect sizes (R^2^ = 0.19 to 0.26) (Table 2). There was no significant association for the therapist-rated alliance (WAI-T) assessed at baseline or at 3-month follow-up with BDI or any of the CORE-OM scales. Moreover, when looking at the change in alliance from the baseline to the 3-month follow-up evaluated by the patients (WAI-P) or therapists (WAI-T), alliance did not significantly predict treatment outcome in any of the outcome measures.

### 3.4. Predicting Treatment Outcome with Congruence in Alliance Ratings between Adolescents and Therapists

When looking at the discrepancy between the adolescent and therapist alliance ratings at baseline (WAI-T minus WAI-P), the associations with CORE-OM total score (*p* = 0.02), CORE-OM well-being (*p* = 0.01), and CORE-OM life functioning (*p* = 0.01) were statistically significant (Table 2). A higher discrepancy was associated with higher scores in the outcome measures. The effect sizes were medium (R^2^ = 0.19 to 0.26).

When the alliance ratings between adolescents and therapists were looked at under four subcategories based on the congruence of the ratings at baseline (1) “weak” by both adolescent and therapist (*n* = 16); (2) “weak” by adolescent and “strong” by therapist (*n* = 13); (3) “strong” by adolescent and “weak” by therapist (*n* = 12); (4) “strong” by both adolescent and therapist (*n* = 16)), the groups differed statistically significantly in CORE-OM total score (*p* = 0.03), CORE-OM well-being (*p* < 0.01), and CORE-OM life functioning (*p* = 0.01) with medium to large effects (R^2^ = 0.25 to 0.31) (Table 2). The group in which adolescents rated the alliance as weak but the therapists rated it as strong (weak–strong group) had worse outcomes in these scales compared with the strong–weak group and with the group where both rated the alliance strong. For CORE-OM well-being, the difference was also significant with the group in which both rated the alliance weak, and the weak–strong group had worse outcomes.

The discrepancy in the ratings at the 3-month follow-up did not predict treatment outcome (*p*-values > 0.05). At the 3-month follow-up, the classified ratings (weak–weak: *n* = 13; weak–strong: *n* = 15; strong–weak: *n* = 13; strong–strong: *n* = 13) were significantly related to poor treatment outcome measured with CORE-OM total score (*p* = 0.01), CORE-OM well-being (*p* = 0.02), CORE-OM problems/symptoms (*p* = 0.03), and CORE-OM life functioning (*p* = 0.01) similarly to at baseline. The “weak–strong” group had significantly higher scores than the “strong–weak” and “strong–strong” groups, but also the “weak–weak” group had significantly higher scores than the adolescents in the “strong–strong” group in all the domains, and it differed significantly also from the “strong–weak” group for CORE-OM total score and CORE-OM life functioning (Table 2). The effect sizes were large (R^2^ = 0.27 to 0.30).

### 3.5. Predicting Treatment Dropout with Alliance

The treatment alliance rated at baseline by the patient (WAI-P) or by the therapist (WAI-T) did not significantly predict treatment dropout (*n* = 10). Low scores in both WAI-P (OR: 0.96; 95% confidence interval (CI): 0.94–0.99; *p* = 0.01) and WAI-T (OR: 0.97; 95% CI: 0.94–1.00; *p* = 0.01) at 3 months was associated with higher risk of dropout from treatment. A change toward lower scores in WAI-P between baseline and the 3-month follow-up was associated with a higher risk of dropout (OR: 0.97; 95% CI: 0.95–1.00; *p* = 0.02). To look at the odds more closely, we analyzed whether change in the alliance across the first 3 months as a binary variable (strengthening alliance or no change vs. weakening alliance) was associated with dropout. Those adolescents reporting a weakening alliance (WAI-P) showed a 4.5 times higher risk (95% CI: 1.02–20.00; *p* = 0.24) of treatment dropout compared with those with strengthening alliance across time or no change in alliance. All effect sizes were small (R^2^ < 0.01). The change in therapist-rated alliance (WAI-T) did not predict treatment dropout. The unbalanced alliance defined by the discrepancy between the adolescent and therapist rating at baseline or at the 3-month follow-up did not significantly predict treatment dropout, nor did the classified ratings of the congruence in the alliance ratings. For the classified patient–therapist alliance ratings at the 3-month follow-up, there were not enough data to conduct the analysis reliably. The relations between alliance ratings and treatment dropout are presented in Table 3.

## 4. Discussion

We conducted the study in order to fill in the gap in current research, which lacks studies using several alliance informants across the treatment of adolescents in a naturalistic setting. Firstly, we examined the difference between adolescent and therapist evaluation of the therapeutic alliance. As hypothesized, and in line with previous studies [19,24], the correlation in the measure of therapeutic alliance was low between the adolescent and the therapist. Moreover, therapists rated the alliance higher throughout the therapy than adolescents, even if the mean difference was not statistically significant. The second aim of the study was to investigate whether and how therapeutic alliance predicted treatment outcome or premature termination of treatment. We found that adolescents’ experience of the strength of the alliance with the therapist is strongly related to the treatment outcome, but therapist assessment did not predict outcome. This means that it is very important not to rely just on the ratings of the therapist regarding the quality of the therapeutic relationship but more importantly to ask the adolescent himself/herself about the alliance. The result is consistent with findings from adult literature [61], but does not support our hypothesis based on previous studies with adolescents [23,33], and does not support the findings from the meta-analysis of Karver et al. (2018) [24], which stated that the alliance perspective does not moderate the relation between alliance and outcome. However, as the meta-analysis of Shirk and Karver (2003) [33], McLeod (2011) [23], and Karver et al. (2018) [24] included methodologically diverse studies involving children and adolescents, and most of the studies reported alliance–outcome relations from only one perspective, it might be argued that it may not be a very firm conclusion to state that the alliance perspective is not significantly associated with the alliance–outcome relation in adolescent psychotherapy. Perhaps because in adolescence, independence from adults increases and the weight of one’s own opinion becomes more important, the adolescent’s evaluation of the strength of the alliance is more crucial for treatment outcome than it was in childhood.

In our study, adolescents who rated better alliance with the therapists at baseline had less severe symptoms and better functioning throughout the treatment in all outcome measures, which is in line with a previous meta-analysis [62] and studies conducted in naturalistic treatment settings with adolescents [20]. However, it should be noted that the effect was found separately for each time point, but there was no interaction with time. This means that it is not possible to conclude from the study that adolescents reporting better alliance would benefit the most from treatment. The relatively weak predictive value might be due to a possible ceiling effect as adolescents, and also therapists, rated the alliance on average quite high. A probable ceiling effect in the WAI has also been reported in previous studies [63]. However, as Karver et al. (2018) [24] highlighted, high ratings on average might suggest that rating of alliance at the midpoint or lower by adolescents should be noted as an alarming sign. One interpretation could also be that the effect of the assessment sessions before the actual therapy started was so significant in terms of early symptom improvement that it affected the perceived alliance at baseline measurement and throughout the treatment [22]. Furthermore, adolescents with less severe symptoms might be more open to new positive relationships or more optimistic about the possibility that the therapist understands their situation and will be able to help. One interesting possible explanation, and also a topic for further research, could be the moderating effect of the attachment of adolescents to their primary caregivers: Zack et al. (2015) [20] found that for adolescents with the poorest attachment history, the working alliance had the strongest relationship with outcome.

The next aim of this study was to investigate whether the strength of the alliance would change over time and whether the change or the timing of the alliance assessment were related to treatment outcome or premature termination of treatment. As expected, we found that the alliance ratings of both adolescents and therapists were quite stable over time. This adds to the previously scarce evidence on the development and change over time of alliance with adolescents [25,64], supports the previous findings that the alliance is formed at an early stage of treatment [22,30,31], and raises the importance of focusing on building the relationship at the very beginning of treatment. According to this finding, the alliance measurement timing would not be of great importance in terms of predicting the treatment outcome. However, contrary to our hypothesis, we found that for the patient-rated alliance, the association with treatment outcome was somewhat stronger at baseline than at 3-month follow-up, suggesting that early assessment might be more predictive. Shirk and Karver (2003) [33] reported larger associations with outcome if alliance was measured late in treatment compared with measures early in treatment, but as the result was based on separate studies and since studies mainly do not include several measuring points for alliance, their result should be interpreted with caution. In our study, the adolescent- and therapist-rated alliances at 3-month follow-up were related to adolescents dropping out from treatment, not the alliance rated at baseline. However, since in our study there were only 10 adolescents who ended the treatment prematurely, the results concerning treatment dropout should be interpreted with caution and be considered only to be setting a benchmark for further studies on the relation of alliance and treatment dropout. Clinically, it is a relevant finding if the alliance is robust over time and assessment from the very beginning of treatment is a significant predictor of outcome. More studies with repeated measures and larger samples are needed to strengthen the conclusions.

We found that change in adolescent- or therapist-rated alliance in the first three months of treatment was not a significant predictor of outcome. Our findings are not in line with earlier studies emphasizing change in alliance ratings [21] and even suggesting that change in alliance ratings could be even more predictive than early alliance itself in adolescents [34,35]. This might be because in our study, the change in alliance over the course of treatment was not statistically significant. Yet, a change for the worse in adolescent-rated alliance in the first three months predicted treatment dropout by the 12-month follow-up. Our study supports the findings from O’Keeffe et al. (2020) [36], who also stated that change in alliance would be more predictive with respect to premature termination of treatment than early alliance. In clinical practice but also in research, young adolescents are not always used as informants, with decisions regarding treatment instead relying on the evaluation and recommendations from adults—professionals and parents [37]. This could result in alliance ruptures not standing out and being processed in therapy, which might be one explanation why adolescents seem to drop out from therapy more frequently than adults [37]. Ulberg et al. (2021) [1] concluded that if therapists focus on the patient–therapist relationship during treatment, explore the patient’s thoughts and feelings toward the therapist, and negotiate the relationship, this increases symptom reduction. Problems in the therapeutic relationship is the most frequently mentioned reason for premature termination of treatment in youths [25].

So far, there has been very little research in which the alliance assessments from several informants have been taken into account at the same time, especially regarding the alliance ratings from adolescents and therapists. To our knowledge this is only the second study aiming to look at this relation. As we hypothesized, the discrepancy between the alliance ratings of adolescents and therapists at baseline predicted treatment outcome. The larger the discrepancy, the poorer the outcome in CORE-OM total score, well-being, and life functioning. Following van Benthem et al. (2020) [5], we conducted a median split in the alliance measured by the adolescents and the therapists. In the resulting four groups (strong–strong, strong–weak, weak–strong, weak–weak), we found similar effects at baseline and at the 3-month follow-up: when the adolescent rated the alliance weak but the therapist rated it strong, the outcome of the therapy was the poorest. However, at the 3-month follow-up, the group where both adolescent and therapist rated the alliance as weak also resulted in poor treatment outcome. This suggests that if the therapist is aware of the discrepancy in the experienced alliance at the beginning of the treatment and is able to work on the relationship, the outcome might be good, but if after the first three months of therapy both the patient and the therapist agree that the alliance is weak, it is especially important to focus on issues that impact the alliance negatively in order for the adolescent to gain from the treatment.

In summary, our findings suggest that it is highly recommended to use both adolescents and therapists as informants of therapeutic alliance, and the alliance should be measured repeatedly, as early alliance and alliance measured later in the treatment predict the outcomes and premature treatment termination differently, and the change in patient-rated alliance is also important to capture. Therapists should actively discuss with adolescents the therapeutic relationship and utilize the measurement instruments as therapeutic tools helping to guide the treatment in order to detect the possible incongruences in the perceived alliance and improve the relationship with the adolescent to gain more positive outcomes.

### Strengths and Limitations

A strength of this study is using several time points for measuring alliance, allowing us to study the constancy of perceived alliance during the treatment and also to compare the association between alliance and treatment outcome or dropout at the beginning of and during the treatment. A significant strength is also the use of both adolescents and psychotherapists as informants of the alliance, as to our knowledge, this study is the first after van Benthem et al. (2020) [5] not just comparing but also combining the evaluations of alliance from adolescents and psychotherapists in order to predict the outcome and premature termination of treatment. The naturalistic setting with clinically referred adolescents is also a strength, as it makes our results more generalizable to clinical practice.

Limitations of the study include a relatively small sample size. The number of participants limits the statistical power in the analyses and increases the risk that some of the results might be caused by type 1 error. Especially for the classified patient–therapist alliance, the number of patients in each of the four groups was low, making the results tentative, so further studies with larger sample sizes are needed. The heterogeneity of the sample might cause more variance in the measured variables. Moreover, as in our study the number of dropouts was very low, the results related to premature termination of treatment should be interpreted very carefully and mostly be considered as setting a benchmark for further studies on the alliance–dropout relation. Despite adjusting for the effects of age, gender, and medication use in the analysis, the effect of other possible confounders or moderators, such as baseline symptom severity or symptom improvement prior to treatment, cannot be excluded. In addition, as treatments were not randomized and there was no control group, we cannot make any causal inferences.

All the measures of our study are widely used and properly validated, but for the outcome assessment the study relied only on self-reported outcome measures. Further, the alliance was measured with an instrument (WAI) that was originally created for adults. Even though WAI is largely used also with adolescents, the three-factor structure and measurement properties may not be generalizable to youth populations [21]. With this in mind, we assumed a single-factor solution as recommended in previous studies with adolescents [27,28,29]. The baseline alliance was measured after the assessment sessions (1–3), so it is possible that the results are confounded with those due to the effect of early symptom improvement on the perceived therapeutic alliance at baseline.

In this study, the parent–therapist alliance was not measured. This alliance could moderate the relation between adolescent alliance and outcome [31]. It has also been stated that parent-rated alliance would be a better predictor of treatment persistence, while youth-rated alliance would be a better predictor of symptom reduction [25,65]. However, the relation between therapist–parent alliance and outcome has been found to be about the same magnitude as the therapist–patient alliance in youth psychotherapy [24]. In addition, this study does not report the patient-related predictors of the working alliance that have been found to be a particularly important focus in the research on alliance [66]. In addition, it should be noted that this study did not assess the moderators (e.g., client-, therapist-, or treatment-related variables) of the alliance–outcome relation. Even positive alliance may not predict successful treatment if certain variables (e.g., problem type, referral source, timing of alliance assessment) are present [19]. Type of treatment might also be a significant moderator of the alliance–outcome relation [22]. These would be prominent research questions for further studies.

## 5. Conclusions

The first sessions in psychotherapy, or even the assessment meetings before therapy, are very important in the formation of the alliance between adolescents and therapists, as the experienced level of alliance does not seem to change over the course of therapy. Therapists are not very good informants of the therapeutic alliance in adolescent psychotherapy, as adolescents seem to evaluate the relationship quite differently. For predicting treatment outcome, the early assessment of adolescent-rated alliance is the most informative. In addition, discrepancy between the perceived alliance of adolescent and therapist both at early alliance assessment and an assessment later in treatment is an important warning sign of unsuccessful treatment. For predicting premature termination of treatment, the alliance ratings later in treatment are more significant than the alliance measured at the very beginning of treatment. In particular, a change for the worse in the adolescent-rated alliance should be considered alarming.

If therapists used the assessments of a therapeutic relationship as a frequent tool within therapy, it might benefit the therapist as he/she could pay special attention to the situations when the adolescent feels the alliance is weakening or his/her experience of the alliance is not in line with the therapist’s. Therapists could focus on improving the relation, which would more likely lead to the adolescent committing to the treatment and gaining a better outcome in therapy. As Horvath et al. (2011) [16] emphasize, alliance development is a skill that therapists can and should be trained in equally to other aspects of giving treatment.

## Figures and Tables

**Table 1:** Alliance ratings at different time points.

Assessment Point		WAI-P	WAI-T	r
Baseline	*n*	57	54	
	Mean	186.20	195.65	0.09
	SD	31.60	22.73	
	Median	186	196	
	Range	105.88–246.00	138.71–252.00	
3-month follow-up	*n*	57	56	
	Mean	189.26	192.65	0.24
	SD	35.20	27.95	
	Median	190.00	195.50	
	Range	96.00–245.83	119.00–252.00	
6-month follow-up	*n*	46	52	
	Mean	191.56	195.43	0.16
	SD	32.32	23.50	
	Median	191.76	195.50	
	Range	123.00–248.00	140.00–249.00	
12-month follow-up	*n*	39	42	
	Mean	194.02	203.16	0.13
	SD	39.35	19.32	
	Median	192.00	204.50	
	Range	64.00–248.00	166.91–252.00	

Note. SD, standard deviation; WAI-P, Working Alliance Inventory, Patient; WAI-T, Working Alliance Inventory, Therapist; r, Pearson correlation.

**Table 2:** Treatment outcome predicted by alliance ratings.

	BDI Total Score						CORE-OM Total Score						CORE-OM Well-Being						CORE-OM Problems/Symptoms						CORE-OM Life Functioning						CORE-OM Risk/Harm					
	*df1*	*df2*	*F*	*p*	R²	Obs. Power (%)	*df1*	*df2*	*F*	*p*	R²	Obs. Power (%)	*df1*	*df2*	*F*	*p*	R²	Obs. Power (%)	*df1*	*df2*	*F*	*p*	R²	Obs. Power (%)	*df1*	*df2*	*F*	*p*	R²	Obs. Power (%)	*df1*	*df2*	*F*	*p*	R²	Obs. Power (%)
Patient alliance rating (WAI-P) at baseline	1	52.64	6.19	0.016 *	0.25	44	1	52.37	9.34	0.004 *	0.25	80	1	51.93	9.56	0.003 *	0.29	72	1	52.13	8.40	0.005 *	0.22	70	1	52.02	9.54	0.003 *	0.23	85	1	54.34	4.02	0.050 *	0.12	34
Patient alliance rating (WAI-P) at 3-month follow-up	1	52.40	2.60	0.113	0.22	20	1	51.13	5.64	0.021 *	0.22	57	1	51.65	6.18	0.016 *	0.26	42	1	51.54	4.15	0.047 *	0.19	45	1	50.25	6.75	0.012 *	0.21	89	1	55.08	1.63	0.207	0.10	13
Therapist alliance rating (WAI-T) at baseline	1	52.26	0.11	0.745	0.18	22	1	52.22	0.76	0.387	0.14	7	1	52.39	0.57	0.455	0.19	7	1	51.75	0.88	0.352	0.13	8	1	52.01	0.89	0.350	0.12	9	1	54.06	0.13	0.722	0.07	6
Therapist alliance rating (WAI-T) at 3-month follow-up	1	53.14	0.79	0.379	0.19	48	1	53.34	0.01	0.933	0.15	35	1	54.07	<0.01	1.000	0.21	54	1	52.56	<0.01	0.950	0.14	18	1	53.41	0.03	0.872	0.12	5	1	55.72	0.04	0.834	0.08	20
Change in patient alliance rating from baseline to 3-month follow-up	1	52.94	0.07	0.794	0.20	7	1	54.04	0.01	0.929	0.17	10	1	55.20	0.01	0.912	0.22	5	1	53.47	0.02	0.895	0.15	5	1	53.63	0.06	0.814	0.15	4	1	56.48	0.03	0.861	0.09	13
Change in therapist alliance rating from baseline to 3-month follow-up	1	52.13	3.71	0.059	0.22	26	1	52.91	2.26	0.139	0.18	48	1	54.07	1.47	0.230	0.22	43	1	52.09	2.63	0.111	0.18	43	1	53.14	2.67	0.108	0.15	34	1	55.26	0.12	0.731	0.09	28
Discrepancy in patient–therapist alliance ratings at baseline	1	50.97	2.77	0.102	0.21	7	1	51.54	5.54	0.022 *	0.19	11	1	51.75	8.07	0.006 *	0.26	25	1	51.23	2.87	0.096	0.16	9	1	51.24	8.74	0.005 *	0.19	37	1	52.30	2.02	0.162	0.10	5
Discrepancy in patient–therapist alliance ratings at 3-month follow-up	1	49.24	0.35	0.557	0.19	38	1	49.14	0.29	0.590	0.15	4	1	49.34	0.37	0.544	0.20	9	1	48.96	0.28	0.598	0.14	6	1	48.86	0.43	0.517	0.12	3	1	50.58	0.13	0.717	0.09	44
Classified patient–therapist alliance ratings at baseline	3	49.31	2.25	0.094	0.25	46	3	49.83	3.27	0.029 *	0.25	82	3	49.35	4.30	0.009 *	0.31	3	3	49.49	2.31	0.088	0.20	56	3	49.52	4.24	0.010 *	0.25	96	3	51.15	1.33	0.274	0.13	26
Classified patient–therapist alliance ratings at 3-month follow-up	3	46.37	2.44	0.077	0.28	72	3	45.28	3.95	0.014 *	0.29	88	3	45.57	3.47	0.024 *	0.30	90	3	45.73	3.14	0.034 *	0.27	78	3	44.70	4.65	0.007 *	0.28	99	3	47.25	1.55	0.214	0.14	49

Obs. Power, observed power; WAI-P, Working Alliance Inventory, Patient; WAI-T, Working Alliance Inventory, Therapist. * Significant at *p* < 0.05 level.

**Table 3:** Treatment dropout predicted by alliance ratings.

	*p*	OR	95% CI for OR	R²	Obs. Power (%)
Patient alliance rating (WAI-P) at baseline	0.190	0.99	0.96–1.01	<0.01	26
Patient alliance rating (WAI-P) at 3-month follow-up	0.009 *	0.96	0.94–0.99	<0.01	93
Therapist alliance rating (WAI-T) at baseline	0.257	0.98	0.95–1.02	<0.01	21
Therapist alliance rating (WAI-T) at 3-month follow-up	0.011 *	0.97	0.94–1.00	<0.01	69
Change in patient alliance rating from baseline to 3-month follow-up	0.021 *	0.97	0.95–1.00	<0.01	69
Change in therapist alliance rating from baseline to 3-month follow-up	0.077	0.97	0.94–1.00	<0.01	37
Discrepancy in patient–therapist alliance ratings at baseline	0.945	1.00	0.97–1.04	<0.01	5
Discrepancy in patient–therapist alliance ratings at 3-month follow-up	0.908	1.00	0.97–1.03	<0.01	5
Classified patient–therapist alliance ratings at baseline	0.823				
weak–weak	0.626	1.63	0.23–11.46	0.02	27
weak–strong	0.877	1.18	0.14–9.83	<0.01	7
strong–weak	0.381	2.44	0.33–17.91	0.06	39
strong–strong (reference group)					
Classified patient–therapist alliance ratings at 3-month follow-up	0.935				
weak–weak	0.999	7.18 × 10^8^	n/a	0.95	0
weak–strong	0.999	4.04 × 10^8^	n/a	0.96	0
strong–weak	0.999	5.38 × 10^8^	n/a	0.96	0
strong–strong (reference group)					

Note. CI, confidence interval; Obs. Power, observed power; OR, odds ratio; WAI-P, Working Alliance Inventory, Patient; WAI-T, Working Alliance Inventory, Therapist. * Significant at *p* < 0.05 level.

## Data Availability

Data sharing not applicable.
